# Mycoplasma penetrans bacteremia and primary antiphospholipid syndrome.

**DOI:** 10.3201/eid0501.990122

**Published:** 1999

**Authors:** A Yáñez, L Cedillo, O Neyrolles, E Alonso, M C Prévost, J Rojas, H L Watson, A Blanchard, G H Cassell

**Affiliations:** Centro de Investigación Biomédica de Oriente-IMSS, Puebla City, Mexico. ayanez@gemtel.com.mx

## Abstract

Mycoplasma penetrans, a rare bacterium so far only found in HIV-infected persons, was isolated in the blood and throat of a non-HIV-infected patient with primary antiphospholipid syndrome (whose etiology and pathogenesis are unknown).


### *Mycoplasma penetrans* Bacteremia and Primary Antiphospholipid Syndrome[1]

**Antonio Yáñez,* Lilia Cedillo,† Olivier Neyrolles,‡ Encarnación Alonso,* Marie-Christine Prévost,‡ Jorge Rojas,* Harold L. Watson,§ Alain Blanchard,‡ and Gail H. Cassell§**

*Centro de Investigación Biomédica de Oriente-IMSS, Puebla City, Mexico; †Benemérita Universidad Autónoma de Puebla, Puebla City, Mexico; ‡Institut Pasteur, Paris, France; and §University of Alabama at Birmingham, Birmingham, Alabama, USA

*Mycoplasma penetrans*, a rare bacterium so far only found in HIV-infected persons, was isolated in the blood and throat of a non-HIV-infected patient with primary antiphospholipid syndrome (whose etiology and pathogenesis are unknown).

Antiphospholipid syndrome (APS), first described in 1983 to 1986, is characterized by a wide variety of hemocytopenic and vaso-occlusive manifestations and is associated with antibodies directed against negatively charged phospholipids. Features of APS include hemolytic anemia, thrombocytopenia, venous and arterial occlusions, livedo reticularis, pulmonary manifestations, recurrent fetal loss, neurologic manifestations (stroke, transverse myelitis, Guillain-Barré syndrome); and a positive Coombs test, anticardiolipin antibodies, or lupus anticoagulant activity (1). The factor(s) causing production of the antiphospholipid antibodies in primary antiphospholipid syndrome (PAPS) remain unidentified (2).

A substantial number of patients with *Mycoplasma pneumoniae*–induced respiratory disease have anticardiolipin antibodies (3). Furthermore, many clinical criteria for APS have also been well documented in patients with *M. pneumoniae* infection, including Guillain-Barré–like illness and other central nervous system manifestations, hemolytic anemia, positive Coombs test, thrombocytopenia, and arthritis (4).

In this report, we describe the case of a patient with clinical features of PAPS and a documented bacteremic infection due to *M. penetrans* (5).

Address for correspondence: Antonio Yáñez, Centro de Investigación Biomédica de Oriente-IMSS, 2º Piso Ala Sur, Hospital de Especialidades, 2 Norte 2004, 72000 Puebla City, Mexico; fax: 52-22-46-00-57; e-mail: ayanez@gemtel.com.mx.

### Case

One month before hospital admission, a previously healthy non-HIV-infected 17-year-old woman (blood group O Rh+) who had not previously received a blood transfusion and had not had sexual experience had acute onset of arthritis of both ankles, generalized arthralgias, fever, progressive asthenia, and hemolytic anemia (hemoglobin 87 g/L, unconjugated bilirubin 25.1 µmol/L). In the 30 days before hospital admission she had not received medications other than nonsteroidal antiinflammatory drugs for 5 days. Three days before hospital admission, she became ill with respiratory distress, generalized weakness, anorexia, and inability to walk.

On admission to the hospital (day 1), physical examination showed severe pallor, a swollen cervical lymph node, slight edema of both legs, tachycardia, and no hypertension. Laboratory data showed severe hemolytic anemia (hemoglobin 34 g/L, lactate dehydrogenase 5.1 µmol/s/L), leukocytosis (24.5 x $10^9$/L), thrombocytopenia (28.0 x $10^9$/L), and normal renal function. A Coombs test was positive at 4ºC, 22ºC, and 37ºC. Blood and bone marrow smears did not show a neoplasic process. The nonsteroidal antiinflammatory drugs were suspended, and treatment was started with a combination of methylprednisolone (1 gm bolus q24h intravenously [i.v.] for 3 days) and trimethoprim/sulfamethoxazole (80/400 mg q12h orally) on day 2, but her condition deteriorated. On day 3, the antibiotic treatment was changed to ceftazidime (1 gm q8h i.v.).

[1]Presented in part at the 11th International Congress of the International Organization for Mycoplasmology. July 14–19, 1996. Orlando, FL, USA.

Transfusion was not attempted because serologic tests indicated the lack of compatibility; (there was a strong positive mismatch incompatibility in 55 different blood samples and a mild mismatch in one sample). Another transfusion was partially rejected because of unidentified nonspecific antibodies. On day 4 severe respiratory distress and hypoxemia developed, requiring a ventilator, and the patient was admitted to the intensive care unit. Livedo reticularis was noted, and methylprednisolone (125 mg q8h i.v.) was administered. Venereal Disease Research Laboratory tests were negative as were tests for lupus erythematosus. The patient had anti-dsDNA antibodies (Kallestad Quantafluor *Crithidia lucilae* Sanofi Diagnostic Pasteur, Inc.) but positive anticardiolipin antibodies by enzyme-linked immunosorbent assay (ELISA) (100 GPL units) (negative test = <10 GPL units) (ImmunoWell, Cardiolipin Antibody Immunoglobulin [Ig]G ELISA; and Reaads Medical Products, Inc.), which remained positive 4 and 12 months later, and antiplatelet antibodies by immunofluorescence (Anti-Human IgG H-chain Fluorescein conjugated, OTIY-05 Behring Diagnostics). Laboratory data showed hemoglobin 33 g/L, leukocyte 23.6 x $10^9$/L, a prolonged activated partial thromboplastin time >150 seconds (control <42 seconds) and prothrombin time of 26.1 seconds (control 14.0 seconds), International Normalized Ratios value = 3D 2.09, and the presence of lupus anticoagulant (LA) antibodies (prolonged Russell viper venom time and confirmed by the STACLOT LA ELISA test, Reaads Medical Products, Inc.). Respiratory secretions were culture-negative and negative by immunofluorescence for respiratory syncytial virus, adenovirus, influenza A, influenza B, parainfluenza 1,2,3, and *Chlamydia*. Serologic analysis indicated that the patient had no antibodies against HIV, hepatitis B surface and core antigens (HbsAg, HBc), or hepatitis C virus. No acid-fast bacilli or other bacteria were observed on blood and tracheal aspirate smears. In addition, thoracic radiography showed only bilateral diffuse pulmonary infiltrates, which was not suggestive of an anaerobic infection.

On day 2 of hospital admission, blood and throat samples were cultured for aerobic flora and mycoplasma. *M. penetrans* in pure culture was isolated from the patient's blood (isolate HF-1) and throat (isolate HF-3). Later *M. penetrans* was isolated from tracheal aspirate in pure culture (isolate HF-2). Treatment was initiated on day 6 with clindamycin 600 mg q8h i.v. and vancomycin 500 mg q6h i.v. The patient also received transfusion of two units of washed red blood cells.

By the microbroth dilution method (6), the HF-1 isolate was sensitive to clindamycin, clarithromycin, azithromycin, erythromycin, tetracycline, doxycycline, ofloxacin, and chloramphenicol but resistant to vancomycin and gentamicin. After 3 days of treatment, the patient improved clinically and was released from the intensive care unit on day 9; thoracic radiographs were clear.

The unique evidence of thrombosis was a low-degree paresthesia of both legs while the patient was receiving anticoagulant therapy; when the condition developed, anticoagulant therapy was increased. The patient received physiotherapy to correct paresis and reduced sensation in the left foot and ankle region. She left the hospital after 26 days, with minimal evidence of peripheral neuropathy as a sequela.

*M. penetrans* infection was detected in the patient's specimens prior to culture and was confirmed by specific polymerase chain reaction (PCR) (7) (Figure 1A, 1B). Similar results were obtained by another pair of PCR primers also within the 16S rRNA gene and designed for the specific detection of *M. penetrans* (data not

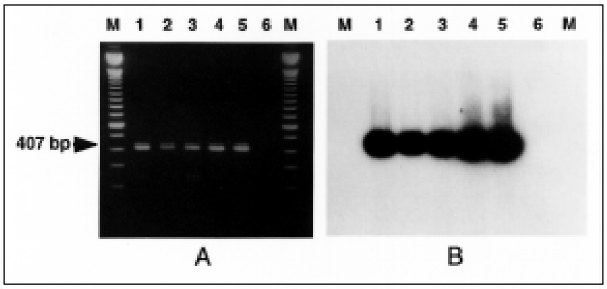

Figure 1. Polymerase chain reaction (PCR) detection of *Mycoplasma penetrans* in clinical samples. A. *M. penetrans* PCR genomic amplification with the primers MYCPENET-P and MYCPENET-N (7) and analyzed by electrophoresis in 2% agarose gel. Lysates from the following original samples: throat swab (lane 1); tracheal aspirate (lane 2); blood (lane 3); first blood subculture (HF-1 isolate) (lane 4); *M. penetrans* GTU-54-6A1 (lane 5), showing the amplification product of 407-bp; and negative control (lane 6). B. Southern blotting of the same material. Hybridization with the internal oligonucleotide (MYCPENET-S) probe confirmed the specific amplification of *M. penetrans* genetic sequences.

shown). Samples from both original specimens and broth cultures were tested by PCR for other human mycoplasmas (8,9), but none were detected (data not shown).

The sodium dodecyl sulfate-polyacrylamide gel electrophoresis (SDS-PAGE) protein patterns of different extracts (whole cell lysate, Triton X-114 extracts) for the isolate HF-1 and the type strain GTU-54-6A1 were almost identical (Figure 2A). Upon close examination, minor differences were found, in particular for antigens approximately 38 kDa in both SDS and Triton X-114 extracts. Ultrastructural examination of the HF isolates by transmission electron microscopy showed mycoplasma cells with morphologic features typical of *M. penetrans* (Figure 2B).

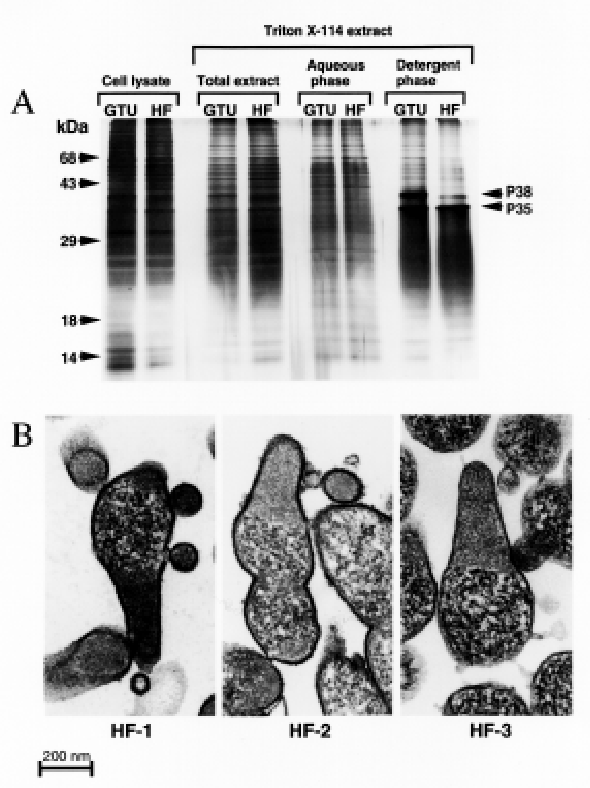

Figure 2. HF isolates belong to the species *Mycoplasma penetrans*. A. Comparison of protein patterns from the type strain of *M. penetrans* and the isolate HF-1. Mycoplasma cells were directly lysed with SDS (cell lysate), or antigens were extracted with the neutral detergent Triton X-114 (total extract). Antigens were further separated after partitioning between the aqueous and detergent phases. The two mycoplasmas compared are the *M. penetrans* type strain GTU-54-6A1 (GTU) and the isolate HF. B. Ultrastructural features of the *M. penetrans* isolates HF-1, HF-2 and HF-3. The HF isolates were passaged four times in SP-4 broth without antibiotics and processed for transmission electron microscopy (TEM). Ultrathin sections were stained with osmiun tetroxide and ruthenium red and observed by TEM. The three isolates show the typical elongated, flask-shaped morphology of *M. penetrans*, with the two divided internal compartments. The cytoplasm is limited by a single triple-layered unit membrane that is covered with capsular material.

Serologic assays (ELISA and Western blot) with Triton X-114-extracted antigens and other *M. penetrans* polypeptides from whole-cell lysates both from the type strain GTU-54-6A1 and from our isolate HF-1 were done as previously described (10). A lack of reactivity against the Triton X-114–extracted antigens of *M. penetrans* was observed by both methods. However, with whole-cell extracts from both type strain and the HF-1 isolate, a 20-kDa polypeptide was immunodetected by Western blotting with three serum samples collected on days 2, 4, and 9 of hospitalization. The 20-kDa polypeptide is an *M. penetrans* product, but whether the observed reaction corresponds to a cross-reacting epitope is not known. The patient's samples were also negative for antibodies against *M. pneumoniae, M. genitalium,* and *M. fermentans* by ELISA (11) (data not shown).

### Conclusions

Since *M. penetrans* was first reported in 1993 as an emerging infectious agent, *M. penetrans*–specific antibodies have been detected more frequently (18.2% to 35.4%) in HIV-infected than in non-HIV-infected persons (0.4% to 1.3%) (10). Until this case, *M. penetrans* had only been isolated eight times (5,10), always from the urine of HIV-infected persons (10).

The results indicating that the isolates HF-1, HF-2, and HF-3 belong to the *M. penetrans* species are as follows: 1) clinical samples and the mycoplasmal isolates obtained from them were positive in the *M. penetrans*-specific PCR assay; 2) protein patterns of the HF isolates and the type strain of *M. penetrans* GTU-54-6A1 were almost identical; 3) serum samples from different patients (10), which contained *M. penetrans*-specific antibodies on the basis of a reaction with the p35 antigen from the type strain of *M. penetrans* also reacted with a similar Triton X-114–extracted polypeptide from the HF-1 isolate; and 4) HF isolates exhibited typical morphologic features of *M. penetrans,* which are unique among mycoplasmas isolated from humans. The fact that serum from other patients reacted with a similar polypeptide from HF isolates indicates that this protein is produced by this strain of *M. penetrans*. The lack of *M. penetrans* strong humoral response in the HF patient was a factor in favor of dissemination of the mycoplasma, hence its isolation from the blood. A possible association between *M. penetrans* and PAPS should be considered.

Snowden et al. (1990) found antiphospholipid antibodies in more than 50% of patients with *M. pneumoniae* pneumonia, especially those with severe infections requiring hospitalization (3). Catteau et al. (1995) described two cases of Stevens-Johnson syndrome associated with *M. pneumoniae* infection and the presence of antiphospholipid antibodies (12). Our patient had manifestations typical of PAPS (2). Thus, this report is the first of *M. penetrans* isolation in a non-HIV-infected patient and the first of a blood and respiratory tract infection with *M. penetrans*.

### Acknowledgments

The authors thank Constantino Gil, M. Ran Nir-Paz, Donna C. Crabb, Lynn B. Duffy, Padma Patel, Blanca Tobón, Angeles Ríos, and Eduardo Aguirre for technical contributions.

This work was supported in part by grant R01 AR 42469, National Institute of Arthritis and Metabolism, National Institutes of Health; the Institut Pasteur; and FOSIZA–CONACYT grant 960202008.

Dr. Yáñez is researcher in the Eastern Biomedical Research Center (Centro de Investigación Biomédica de Oriente), IMSS. He has worked with human mycoplasmas for the last 12 years and is interested in bacterial pathogenesis, in particular in the field of autoimmune diseases triggered by mycoplasmas.

### References

1. Hughes GRV. The antiphospholipid syndrome: ten years on. Lancet 1993;342:341-4.
2. Asherson RA, Cervera R. “Primary,” “secondary,” and other variants of the antiphospholipid syndrome. Lupus 1994;3:293-8.
3. Snowden N, Wilson PB, Longson M, Pumphrey RSH. Antiphospholipid antibodies and *Mycoplasma pneumoniae* infection. Postgrad Med J 1990;66:356-62.
4. Cassell GH, Gray G, Waites KB. Chapter 180: mycoplasmal infections. In: Harrison’s principles of internal medicine. Isselbacher KJ, Braunwald E, Wilson JD, Martin JB, Fauci AS, Kasper DL, editors. Vol. 1, McGraw-Hill Inc, 1997:1052-55.
5. Lo S-C, Hayes MM, Tully JG, Wang RY-H, Kotani H, Pierce PF, et al. *Mycoplasma penetrans* sp. nov., from the urogenital tract of patients with AIDS. Int J Syst Bacteriol 1992;42:357-64.
6. Waites KB, Cassell GH, Cannupp KC, Fernandes PB. In vitro susceptibilities of mycoplasmas and ureaplasmas to new macrolides and aryl-fluoroquinolones. Antimicrob Agents Chemother 1988;32:1500-2.
7. Grau O, Kovacic R, Griffais R, Launay V, Montagnier L. Development of a PCR-based assays for the detection of two human mollicute species, *Mycoplasma penetrans* and *M. hominis*. Mol Cell Probes 1994;8:139-48.
8. Blanchard A, Gautier M, Mayau V. Detection and identification of mycoplasmas by amplification of rDNA. FEMS Microbiol Lett 1991;81:37-42.
9. Blanchard A, Yáñez A, Watson H, Griffiths G, Cassell GH. Evaluation of intraspecies genetic variation within the 16S rRNA gene of *Mycoplasma hominis* and detection by polymerase chain reaction. J Clin Microbiol 1993;31:1358-61.
10. Grau O, Slizewicz B, Tuppin P, Launay V, Bourgeois E, Sagot N, et al. Association of *Mycoplasma penetrans* with immunodeficiency virus infection. J Infect Dis 1995;172:672-81.
11. Cassell GH, Gambil G, Duffy LB. ELISA in respiratory infections of humans. In: Molecular and diagnostic procedures in mycoplasmology. Vol II. Tully JG, Razin S, editors. Academic Press 1996:123-36.
12. Catteau B, Delaporte E, Hachulla E, Piette F, Bergoend H. Infection à mycoplasme avec syndrome de Stevens-Johnson et anticorps antiphospholipides: à propos de deux cas. Rev Med Interne 1995;16:10-4.

